# Machine Learning-Based Calibration of Low-Cost Air Temperature Sensors Using Environmental Data

**DOI:** 10.3390/s17061290

**Published:** 2017-06-05

**Authors:** Kyosuke Yamamoto, Takashi Togami, Norio Yamaguchi, Seishi Ninomiya

**Affiliations:** 1 PS Solutions Corp., 1-5-2 Higashi-Shimbashi, Minato-ku, Tokyo 105-7104, Japan; takashi.togami@g.softbank.co.jp (T.T.); norio.yamaguchi2@g.softbank.co.jp (N.Y.); 2 Graduate School of Agricultural and Life Sciences, The University of Tokyo, 1-1-1 Midori-cho, Nishi-Tokyo, Tokyo 188-0002, Japan; snino@isas.a.u-tokyo.ac.jp

**Keywords:** sensor calibration, low-cost sensor, machine learning, artificial neural network, agriculture

## Abstract

The measurement of air temperature is strongly influenced by environmental factors such as solar radiation, humidity, wind speed and rainfall. This is problematic in low-cost air temperature sensors, which lack a radiation shield or a forced aspiration system, exposing them to direct sunlight and condensation. In this study, we developed a machine learning-based calibration method for air temperature measurement by a low-cost sensor. An artificial neural network (ANN) was used to balance the effect of multiple environmental factors on the measurements. Data were collected over 305 days, at three different locations in Japan, and used to evaluate the performance of the approach. Data collected at the same location and at different locations were used for training and testing, and the former was also used for *k*-fold cross-validation, demonstrating an average improvement in mean absolute error (MAE) from 1.62 to 0.67 by applying our method. Some calibration failures were noted, due to abrupt changes in environmental conditions such as solar radiation or rainfall. The MAE was shown to decrease even when the data collected in different nearby locations were used for training and testing. However, the results also showed that negative effects arose when data obtained from widely-separated locations were used, because of the significant environmental differences between them.

## 1. Introduction

Meteorological monitoring plays an important role in weather forecasting [1], disaster prediction [2], traffic flow prediction [3] and agriculture [4,5,6]. Since climate change has come to be recognized as a serious issue over recent years, meteorological monitoring has gained in importance.

Weather observation systems are installed in many locations throughout the world. The Automated Meteorological Data Acquisition System (AMeDAS) developed by the Japan Meteorological Agency has been installed at 1300 locations in Japan. AMeDAS has sensors to monitor air temperature, humidity, solar radiation/duration, wind speed/direction, rainfall, atmospheric pressure and other factors. Since the accuracy of the data is certified by the Japan Meteorological Business Support Center, data obtained by AMeDAS are used for many purposes including weather forecasting, disaster prediction and crop modeling [7,8,9]. However, such weather observation systems are expensive, and installation is complex. This makes the system challenging to install in many places or other places where measurement is most critical.

To address these problems of cost and flexibility, wireless sensor networks (WSNs) have been developed [10,11,12,13,14,15,16,17]. Since one of the objectives of WSNs is cost reduction, low-cost sensors are often used. However, there is a tradeoff between the accuracy and cost of sensors [18], and the reliability of data acquired by WSNs is often a matter of dispute [19,20,21]. The accuracy of low-cost sensors used for air temperature measurement has been widely studied in agricultural research [19,22,23,20,24,25,26,21,27], because air temperature is an important factor in plant growth.

**Figure 1:**
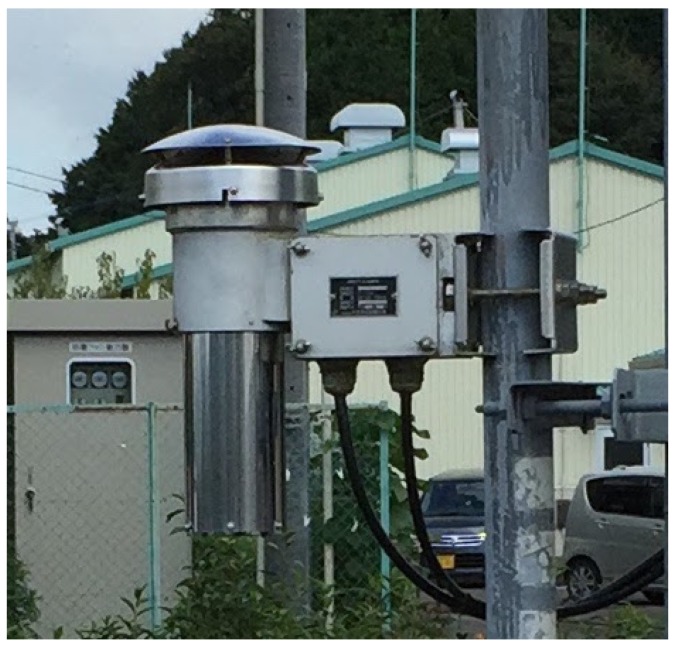
Appearance of the air temperature sensor of the Automated Meteorological Data Acquisition System (AMeDAS). The sensor is covered by a radiation shield made of stainless steel. There is 5-${\mathrm{ms}}^{-1}$ air flow in the radiation shield generated by a forced aspiration system.

Air temperature measurements are influenced by solar radiation, humidity, wind speed, rainfall and the azimuth [19,22,23,26,28]. The effect of solar radiation is the most significant of these. High-accuracy air temperature sensors are therefore covered with radiation shielding made from material with a high heat capacity. Forced aspiration systems are also used to avoid condensation in the cover and to measure the air temperature outside the cover. The air temperature sensors used in AMeDAS (Figure 1) have a forced aspiration system that generates an air speed of 5 ${\mathrm{ms}}^{-1}$. However, these additions make the sensors much more expensive. The forced aspiration system also requires an electric power source, which is often challenging at outdoor sites.

To address this, studies have been conducted with software-based calibration methods for low-cost air temperature sensors. Sun et al. [20] investigated air temperature error correction. They developed a look-up table of solar radiation and the resulting difference in air temperature measured by low-cost and high-accuracy sensors, and they used this to conduct the error correction. They also considered the time lag of temperature measured by low-cost and high-accuracy sensors, caused by the time taken to transmit energy from solar radiation to the atmosphere. Liu et al. [24] investigated the improvement of air temperature measurement by low-cost sensors, using daytime data, which are influenced by the time of sunrise and sunset. The data were preprocessed using discrete wavelet transform to remove noise. They then established a model based on a back propagation neural network, in which the input and output data were air temperature measurements from low-cost sensors and the national ground weather station respectively.

Although these previous studies have improved the accuracy of air temperature measurement by low-cost sensors, there remains considerable room for improvement. First, the data used had very limited variation. Sun et al. [20] used 14 days of data recorded from June to December in the same year, and Liu et al. [24] used the data from 10 cloudy days in June and July of the same year. Seasonal factors are especially critical in Japan, which has four seasons with very different climatic conditions. Therefore, long-term data, measured under these different climatic conditions, are needed to accurately evaluate the performance of calibration. Second, a very limited range of data were used in the previous studies. Sun et al. [20] used air temperature and hourly global solar irradiation, and Liu et al. [24] used only air temperature. It is well known that low-cost air temperature sensors are influenced by the range of environmental factors noted above. Including these in the input data should greatly improve the accuracy of low-cost air temperature sensors.

In this study, we developed a calibration method using long-term air temperature measurement by low-cost sensors and taking account of multiple environmental factors. The data used covered a total of 305 days between January and December, with October data excluded. Data recorded in different parts of Japan were used to investigate the location specificity of our calibration method. To take account of the effect of multiple environmental factors, machine learning was used. Finally, the accuracy of our calibration method was compared with the calibrated air temperature data measured by the high-accuracy sensors of AMeDAS.

## 2. Materials and Methods

### 2.1. Data Acquisition

**Figure 2:**
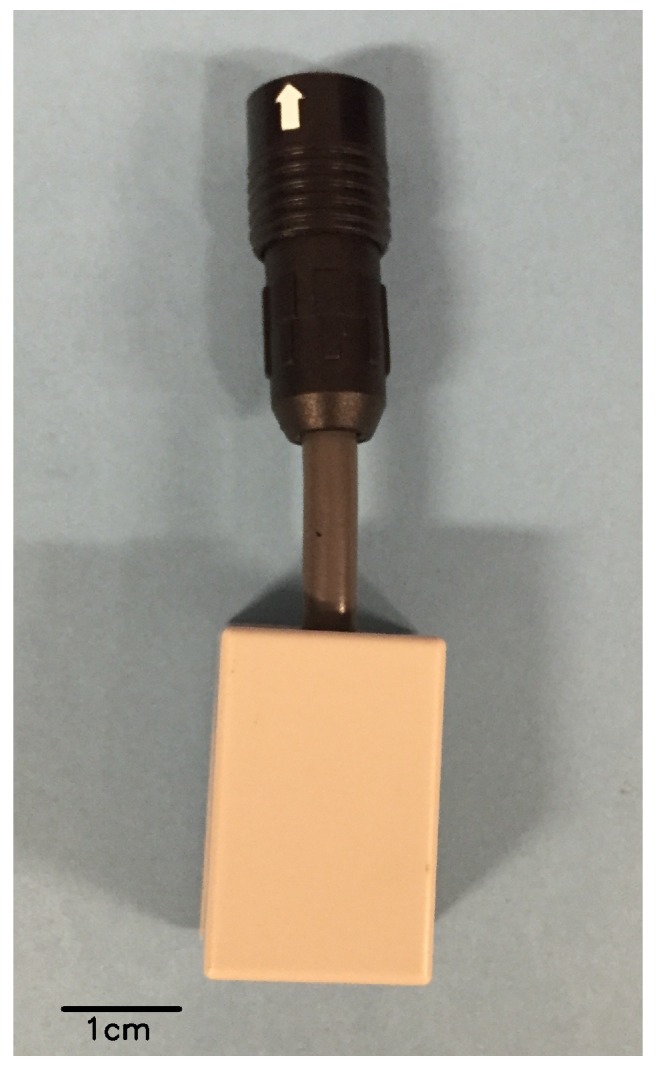
Appearance of the low-cost air temperature sensor.

The low-cost air temperature sensor used in this study is shown in Figure 2. The sensor probe “SHT-71” (Sensirion AG, Staefa, Switzerland) for measuring air temperature and relative humidity is covered by the plastic box. The specification of the sensor probe is given in Table 1. The sensor was connected to a sensor node of the e-kakashi prototype version (PS Solutions Corp., Tokyo, Japan), which consists of ports of air temperature, relative humidity and solar radiation sensors, the GPS unit and the 3G networking module. Measured data were uploaded to our database on Amazon Web Services via the 3G network.

Before the outdoor experiment was conducted, an indoor experiment was carried out to assess the accuracy of the sensor. Low-cost and high-accuracy (reference) sensors were installed inside a building for 15 days to allow the accuracy of the low-cost sensor to be evaluated without the influencing factors of solar radiation, rain or wind. The high-accuracy sensor had a forced aspiration system and radiation shield (CPR-AS-12-AC, Climatec Inc., Tokyo, Japan), and the accuracy of its sensor probe (C-HPT-5-JM, Climatec Inc., Tokyo, Japan) had been certified by the Japan Meteorological Business Support Center. The specification of the sensor probe is given in Table 2.

**Figure 3:**
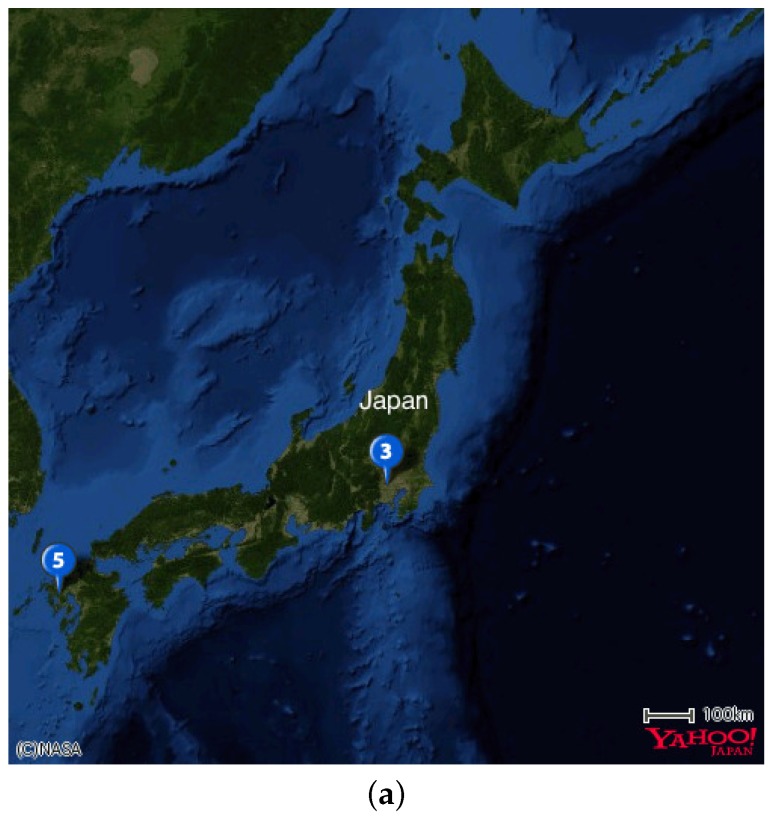

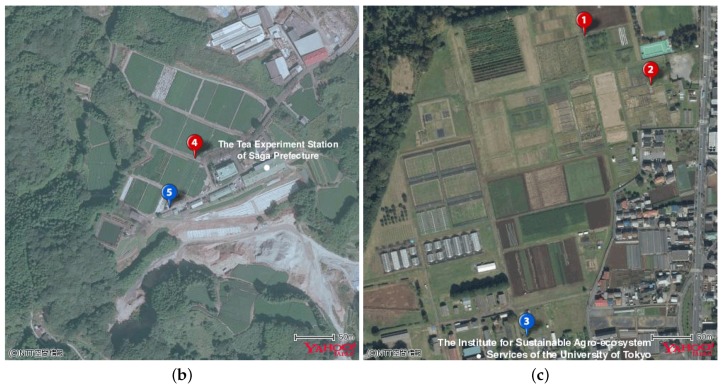
Location map of experimental sites and sensor setups (Pin1: tanashi-117; Pin2: tanashi-118; Pin3: sensor measuring reference air temperature for Tokyo site; Pin4: sagatea-111; Pin5: AMeDAS in Saga site). (**a**) Experimental sites in Japan; (**b**) sensor setup in Saga; (**c**) sensor setup in Tokyo.

**Figure 4:**
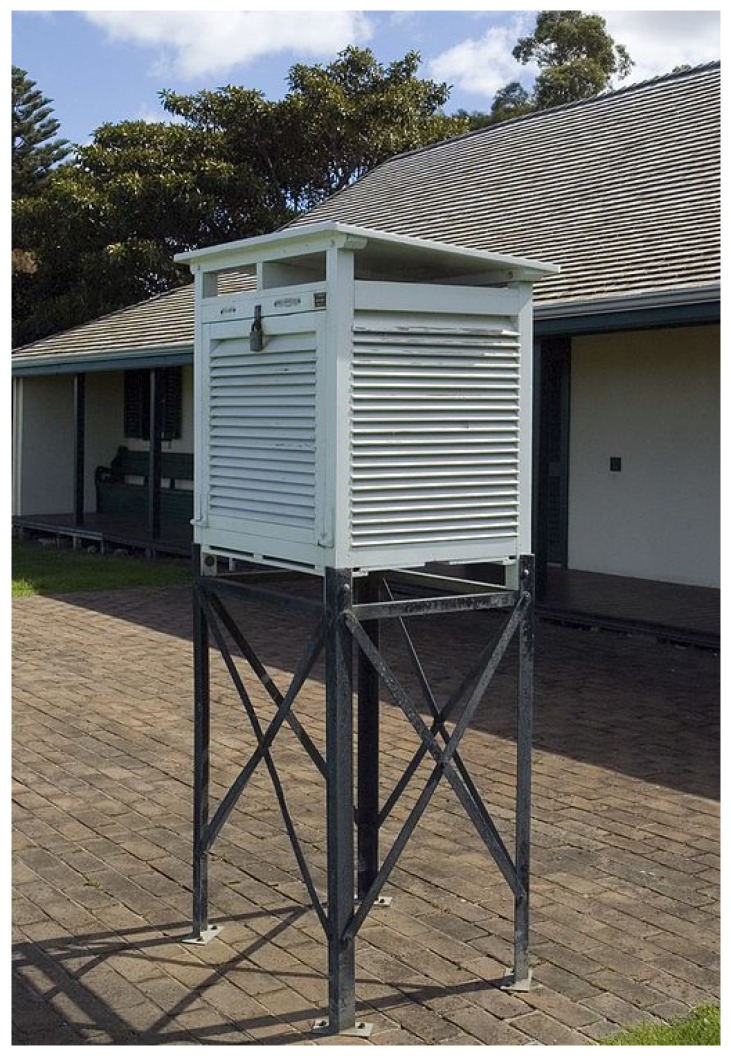
Appearance of Stevenson screens (photographed by Nachoman-au, distributed under a CC BY-SA3.0 license).

The experimental sites and sensor setups are shown in Figure 3. We installed one sensor node in the Tea Experiment Station of Saga Prefecture ($33^{\circ }7^{\prime }3^{\prime \prime }$ N and $129^{\circ }59^{\prime }41^{\prime \prime }$ E) and two sensor nodes in the Institute for Sustainable Agro-ecosystem Services of The University of Tokyo ($35^{\circ }44^{\prime }23^{\prime \prime }$ N and $139^{\circ }32^{\prime }29^{\prime \prime }$ E; $35^{\circ }44^{\prime }20^{\prime \prime }$ N and $139^{\circ }32^{\prime }33^{\prime \prime }$ E). Hereinafter, these locations are denoted as sagatea-111, tanashi-117, and tanashi-118, respectively. Since AMeDAS is also installed at the Tea Experiment Station ($33^{\circ }7^{\prime }1^{\prime \prime }$ N and $129^{\circ }59^{\prime }40^{\prime \prime }$ E), we used the air temperature measurements from AMeDAS as reference data. The AMeDAS data were supplied by MetBroker [8]. At the experimental site in Tokyo, air temperatures measured by sensors installed at $35^{\circ }44^{\prime }9^{\prime \prime }$ N and $139^{\circ }32^{\prime }26^{\prime \prime }$ E were used as reference data. The sensors at the experimental site in Tokyo do not have a forced aspiration system, but are installed in Stevenson screens to shield them from direct sunlight while allowing natural air circulation by shelters made of wood in a double-louvered design (Figure 4).

We used air temperature data taken every hour from November 2014 to September 2015 in Saga and from April 2015 to September 2015 in Tokyo. All of the data collected in this study are available online [29].

### 2.2. Calibration Method

**Figure 5:**
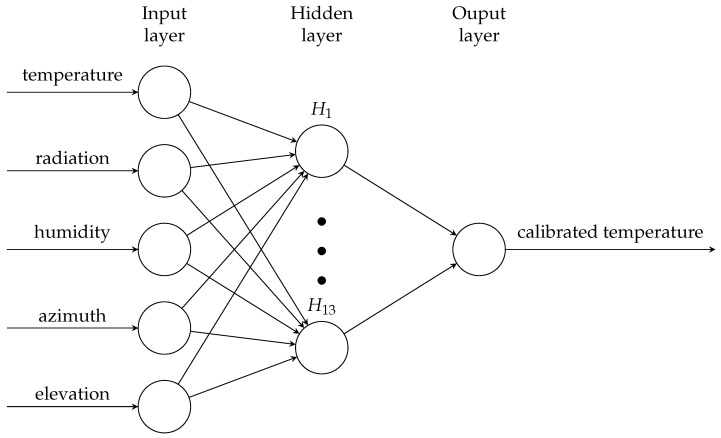
Structure of the neural network.

A three-layer backpropagation artificial neural network (ANN, Figure 5) was used to consider the multiple environmental factors that affect low-cost air temperature sensors. The hidden layer had 13 neurons with the weight decay of 0.1, which were determined by 10-fold cross-validation of datasets obtained at each location. The input data were the air temperatures measured by the low-cost sensor, humidity, solar radiation, azimuth and elevation. The solar radiation (“SP-215”, Apogee Instruments, Inc., Logan, Utah, USA) and humidity (“SHT-71”, Sensirion AG, Staefa, Switzerland) sensors were connected to the sensor nodes, while the solar azimuth and elevation were based on the latitude and longitude obtained by the GPS units on the sensor nodes. Linear regression-based calibration was also conducted to compare the performance of our method with that of a conventional method. All calculations were conducted using R Version 3.3.3 (R Core Team, Vienna, Austria) [30]. Source codes developed for this research are available online [29].

### 2.3. Performance Evaluation

We conducted two types of analysis: within a single location and across the different locations. In the former analysis, we conducted 10-fold cross-validation and repeated this for each location. In the latter analysis, we conducted cross-validation between the locations, using data obtained at one location for training and data obtained at another for testing. This was repeated for all combinations of the three sites, to evaluate the location specificity of our calibration method.

The performance of our calibration method was evaluated using the mean absolute error (MAE) between the air temperatures measured by the low-cost sensors, after calibration, and those measured by the high-accuracy sensors.

## 3. Results

### 3.1. Indoor Experiment

**Figure 6:**
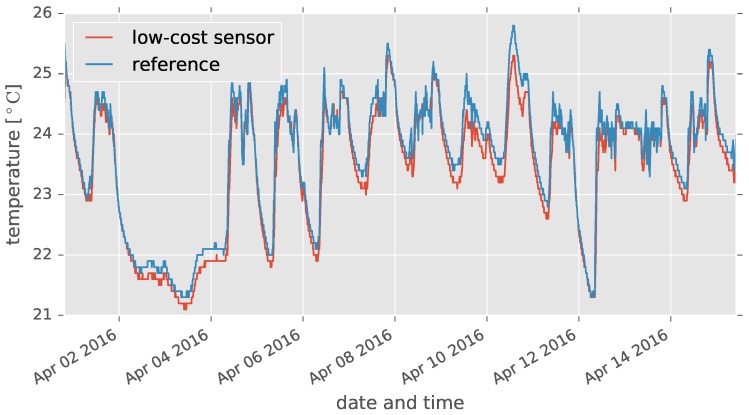
Indoor air temperatures measured by low-cost and high-accuracy (reference) sensors. The indoor experiment was conducted for 15 days, and the air temperatures were very close in the whole period.

Figure 6 shows the indoor air temperatures measured by the low-cost and reference sensors obtained from the indoor experiment. The MAE was 0.19, and the maximum relative error was −1.00.

### 3.2. Cross-Validation within a Location

**Figure 7:**
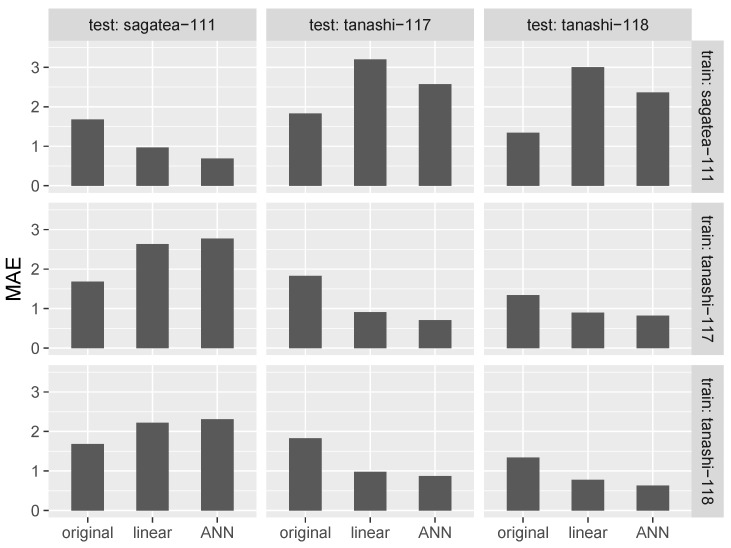
MAE between air temperatures from high-accuracy sensor and low-cost sensor (original), linear regression-based calibration (linear) and ANN-based calibration (ANN).

**Figure 8:**
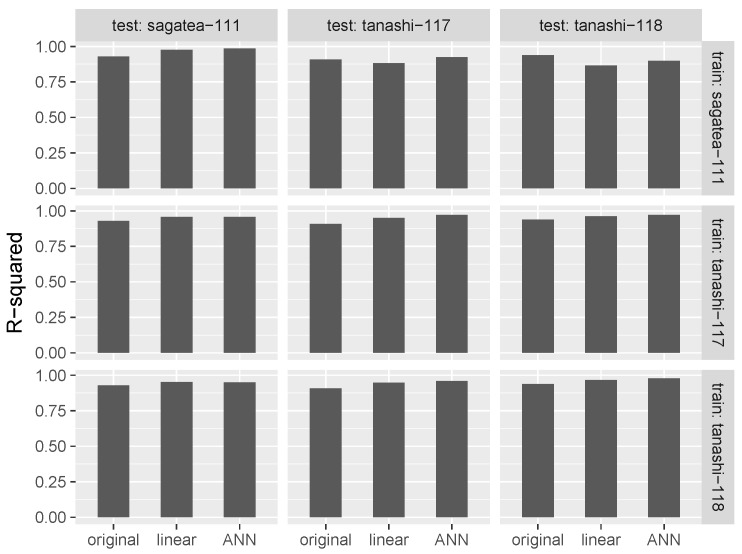
R-squared values between air temperatures from high-accuracy sensor and low-cost sensor (original), linear regression-based calibration (linear) and ANN-based calibration (ANN).

The results of cross-validation are shown in Figure 7 and Figure 8. Diagonal components, for which the training and test locations were the same, represent the results of 10-fold cross-validation within a location. The MAEs and R-squared values were improved by both linear regression-based and ANN-based calibration. ANN calibration performed better at all locations, confirming the effectiveness of our machine learning-based approach.

**Figure 9:**
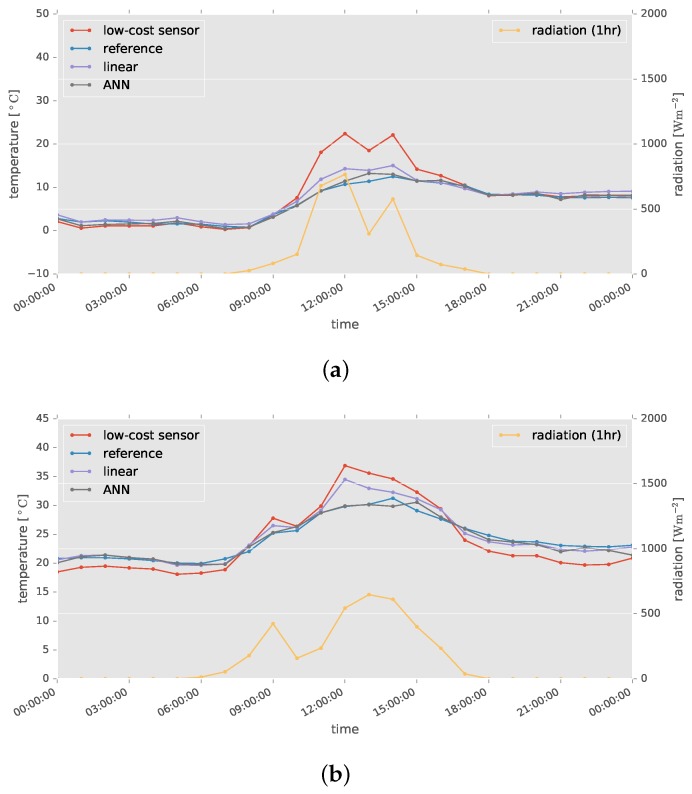
Examples of successful calibration. (**a**) 25 January 2015; (**b**) 28 September 2015.

Example results are shown in Figure 9. The air temperatures measured by the low-cost sensor were clearly higher than those measured by the high-accuracy sensor when solar radiation was strong. These abnormal values were corrected by applying ANN-based calibration, bringing the calibrated air temperatures close to those from the high-accuracy sensors.

**Figure 10:**
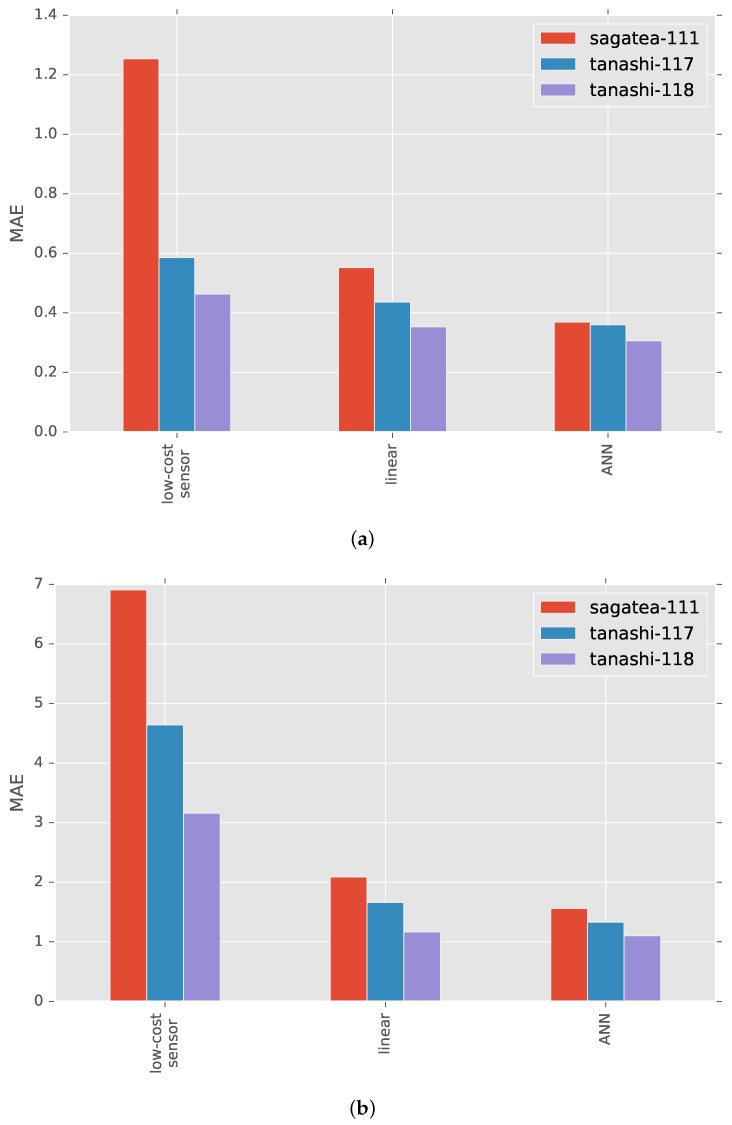
Average and diurnal temperatures before and after calibration. “Low-cost sensor”, “linear” and “ANN” indicate original values, linear regression-based calibration values and ANN-based calibration values of the observations by the low-cost sensors respectively. (**a**) Daily average temperature; (**b**) diurnal range of temperature.

In applications such as crop modeling, daily average temperatures and the diurnal range of temperature are more important than instantaneous data. Figure 10 shows the daily average temperature and diurnal range of temperature before and after calibration. Our method greatly improved both, yielding a further advantage over calibration based on linear regression.

**Figure 11:**
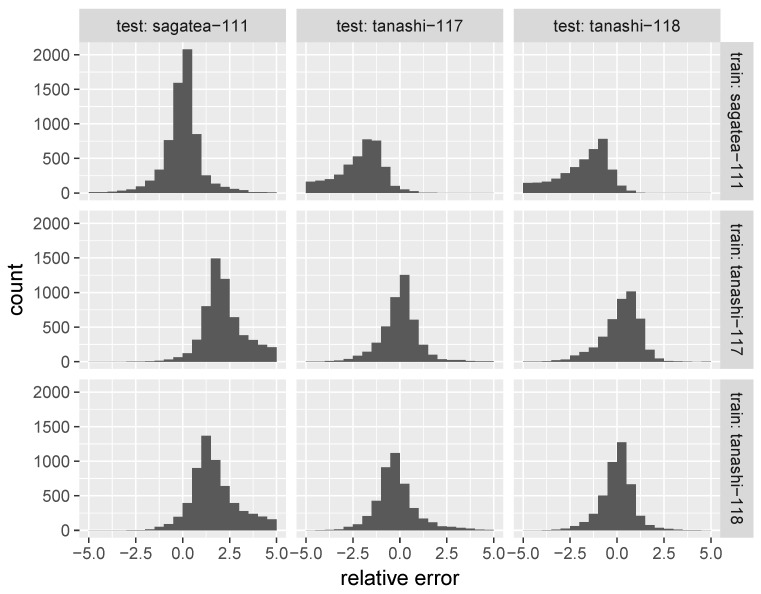
Histogram of relative error between reference and calibrated air temperatures.

**Figure 12:**
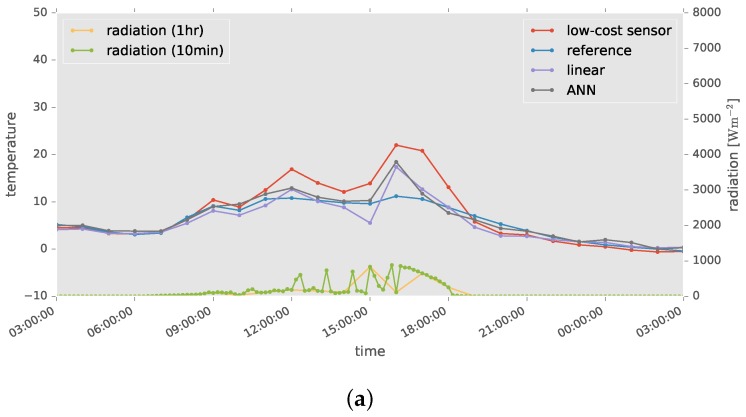

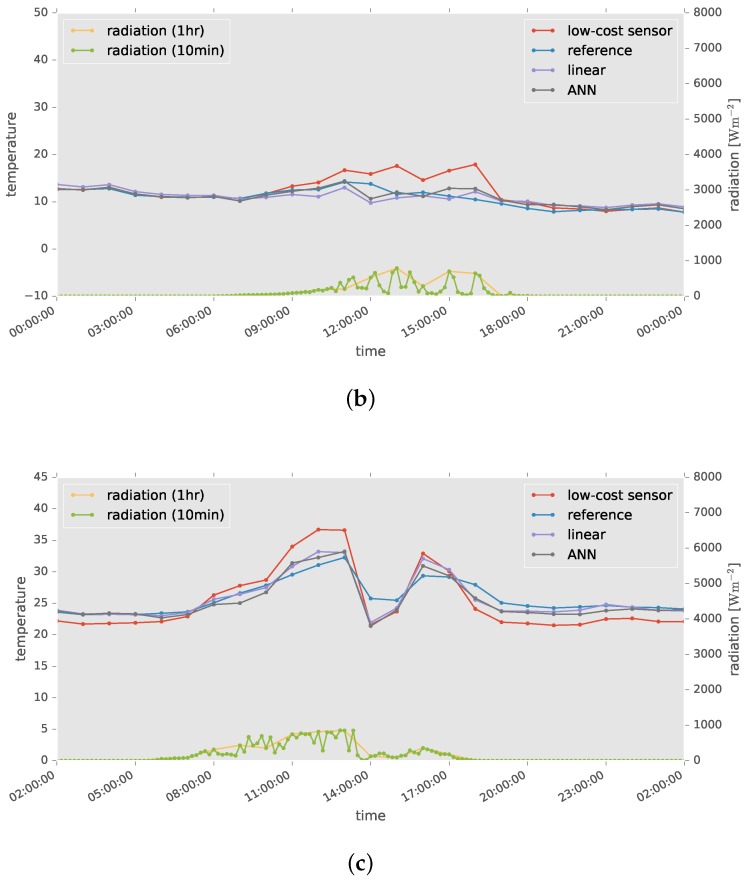
Examples of calibration failure. (**a**) 24 March 2015; (**b**) 14 April 2015; (**c**) 4 September 2015.

Histograms of relative errors between reference and calibrated air temperatures are shown in Figure 11, where diagonal elements represent the result of cross-validation within a location. The histograms peak at a relative error of approximately =0. Some calibration failures are picked up in Figure 12, where solar radiation plotted at 10-min intervals is also shown. The absolute errors became large when the solar radiation, which is the most important factor in outdoor air temperature measurement, changed abruptly.

### 3.3. Cross-Validation between Locations

In Figure 7 and Figure 8, elements other than diagonal ones represent the result of cross-validation between locations. The cross-validation between locations in Tokyo showed that the application of our method resulted in significant improvement in both MAE and the R-squared value. However, negative effects on the MAEs arose when the data used for cross-validation came from the distant locations of Tokyo and Saga, although R-squared values were improved except in the case when sagatea-111 and tanashi-118 were used for training and testing, respectively.

Figure 11 shows the relative error locations. The histograms peak at a relative error of approximately =0 between the locations in Tokyo. The peak becomes more negative or positive for cross-validations conducted at widely-separated locations.

**Figure 13:**
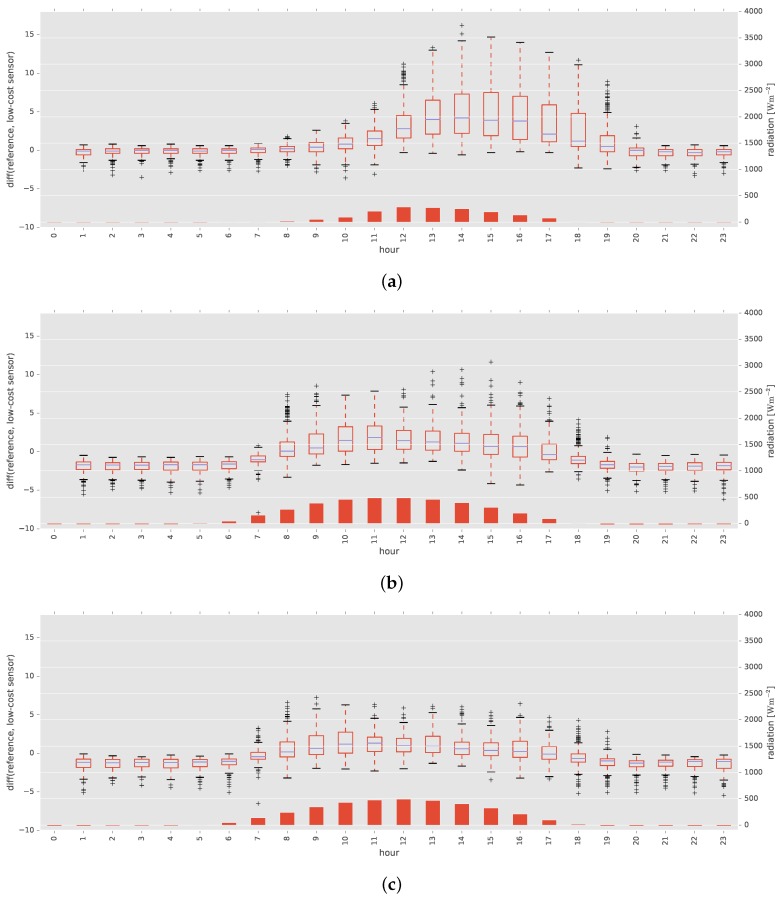
Box plots of differences between air temperatures measured by the high-accuracy and low-cost sensors at different hours. Bar plots represent mean solar radiation in each hour. (**a**) sagatea-111; (**b**) tanashi-117; (**c**) tanashi-118.

Figure 13 represents the variation in relative errors between air temperatures measured by the low-cost and high-accuracy sensors. Although the variations were similar at the two locations in Tokyo, there were obvious differences between Tokyo and Saga.

## 4. Discussion

This study proposed and investigated a calibration method for correcting the air temperature measurements produced by low-cost sensors under natural conditions. We used a machine learning approach to allow the effect of multiple environmental factors to be considered, including humidity, solar radiation, azimuth and elevation. These are known to negatively affect the accuracy of low-cost air temperature sensors. We first conducted an indoor experiment to confirm the accuracy of the low-cost air temperature sensor under ideal conditions. We then conducted two further analyses: calibration using data measured at a single location and calibration using data measured at nearby and distant locations in Japan. As shown in Figure 13, there were obvious differences in climatic environments between the experimental sites, indicating the need to evaluate calibration methods with data collected at multiple locations, which has not been conducted in the related research.

The results of the indoor experiment (shown in Figure 6), conducted under ideal conditions without any influence from factors, such as solar radiation, rain and wind, confirmed that the low-cost sensor met the specifications shown in Table 1. We concluded that any measurement errors arising in the outdoor experiments could be mainly attributed to environmental factors.

The results from the cross-validation within a single location confirmed that the accuracy of the low-cost sensor was considerably improved by the proposed method, compared with the traditional method based on linear regression. However, some large errors occurred, as shown in Figure 11. Some of them are shown in Figure 12. For example, Figure 12a shows solar radiation decreasing rapidly at 16:00, before rising again. This may have been caused by a cloud briefly hiding the Sun. The model decreased the observed temperature by a small amount, as the input solar radiation was also low. On the other hand, in the case of Figure 12b, solar radiation increased rapidly at 12:00, and the observed temperature was therefore greatly decreased by our model. However, as solar radiation had been low between 11:30 and 11:50, the observed temperature was close to the reference value, and the calibration failure shown in Figure 12b occurred. The proposed model used only the environmental conditions of the time when the temperature to be calibrated was observed and did not consider the preceding environmental conditions, which could also cause the observation errors. Therefore, a particularly rapid change of environmental conditions tends to result in the failure of the calibrations. In order to solve the issue, a future study needs to include preceding environmental conditions in the ANN model, discovering the most appropriate period of prior time series observations. We also expect that the higher observation frequency may improve the calibration accuracy.

There was another cause of calibration failures. In Figure 12c, the air temperatures measured by both the high-accuracy and low-cost sensors drastically decreases at around 14:00. A nearby AMeDAS station, located at $35^{\circ }44^{\prime }$ N and $139^{\circ }35^{\prime }$ E, or approximately 3.9 km from the experimental site in Tokyo, observed sudden heavy rain at this time. It is likely that heavy rain also occurred at our experimental site, producing a rapid decrease in air temperature. As our model does not consider rain levels or wind speed, calibration may have failed. Since we used an ANN for the calibration model, it is expected to be easy to add other environmental factors in a future study if the observations are available.

The results from the cross-validation between locations showed that the R-squared values with reference temperature were improved in most cases. The results also showed that MAEs were improved when the data collected in different nearby locations were used. However, negative effects on MAEs arose when data obtained from widely-separated locations were used. The difference in variation at each hour, shown in Figure 13, was considered the possible cause. This difference in variation reflects the different environments of Tokyo and Saga prefectures. For example, the solar radiation at 15:00 in Saga and 16:00 in Tokyo was very similar, but the variation in temperature was clearly different. Our method performed poorly when data obtained from totally different environments were used for training. These results suggest the need to prepare datasets obtained from a range of environments and a dataset measured under similar environments as a test case in practical applications. Even though such data collection requires time and labor costs, long-term cost should be less than using expensive high-accuracy sensors. This is because, once training data are collected at a location, the same model can be applied even when the low-cost sensor is broken and renewed at the location. Our approach also has an advantage over expensive high-accuracy sensors in terms of flexibility; the combination of our approach and low-cost sensors without the forced aspiration system does not require a large amount of electric power, which is often a challenge for use at outdoor sites, but achieves higher accuracy.

The calibration results showed that our method also improved the daily average temperature and the diurnal range of temperature. These are especially important values in crop modeling, because the accumulated value of the former is used as the index of flowering and harvesting, and the latter indicates the balance between photosynthesis and transpiration. Therefore, we expect that our method has the ability to improve the accuracy of crop modeling. Significant research has been conducted on sensor observation systems, to ensure interoperability among different WSNs. One of the objectives of such research is to make data on a range of climatic factors, measured by sensors of different accuracy in different parts of the world, accessible to improve crop modeling. However, as shown in this study, data measured by low-cost sensors are less reliable. Research is needed both on the acceleration of data accessibility and improvement of data accuracy, to drive data utilization in agricultural studies.

This study calibrated the air temperatures measured by low-cost sensors using a software-based approach. Several hardware-based approaches are available for improving the accuracy of low-cost sensors, such as the use of radiation shielding or heat-resistant coatings. The performance of these has already been demonstrated [19,21,22,23,25,26,27,31]. In future studies, a combination of software- and hardware-based approaches may be demonstrated to offer further improvements in accuracy.

## 5. Conclusions

A new calibration method for correcting the air temperature measured by low-cost sensors under natural conditions was proposed. We used an artificial neural network (ANN) to balance the effect of multiple environmental factors on the air temperature measurements. Data were collected over 305 days, at three different locations in Japan, and used to evaluate the performance of the method. As a preliminary experiment, we conducted an indoor experiment and confirmed that any measurement errors arising in the outdoor experiments could be mainly attributed to environmental factors. We then conducted two further analyses; calibration using data measured at a single location and calibration using data measured at nearby and distant locations. The results of these analyses showed that our method works well when training and test data were collected under similar environments, even if they were different locations. However, there was a negative effect when data from to distant locations, where environmental variations were totally different, were used. In a future study, we need to consider preceding environmental conditions, observation frequencies and other environmental information such as rain levels and wind speed to improve our method. We also need to prepare datasets obtained from a range of environments for the practical applications in future work.

## Figures and Tables

**Table 1:** Specification of the sensor probe of the low-cost air temperature sensor.

Parameter	Value (°C)
Measuring range	−40 to 123.8
Resolution	0.01
Accuracy	±0.5 (25 °C)
	±0.9 (0 to 40 °C)

**Table 2:** Specification of the sensor probe of the high-accuracy sensor for the indoor experiment.

Parameter	Value (°C)
Measuring range	−50 to 50
Resolution	0.1
Accuracy	±0.15+0.002|reading|
